# What Can We Learn From Others to Develop a Regional Centre for Infectious Diseases in ASEAN? Comment on "Operationalising Regional Cooperation for Infectious Disease Control: A Scoping Review of Regional Disease Control Bodies and Networks"

**DOI:** 10.34172/ijhpm.2022.7281

**Published:** 2022-06-14

**Authors:** Yot Teerawattananon, Saudamini Vishwanath Dabak, Wanrudee Isaranuwatchai, Thongchai Lertwilairatanapong, Asrul Akmal Shafie, Auliya A. Suwantika, Cecilia Oh, Jaruayporn Srisasalux, Nopporn Cheanklin

**Affiliations:** ^1^Health Intervention and Technology Assessment Program (HITAP), Ministry of Public Health, Nonthaburi, Thailand.; ^2^Saw Swee Hock School of Public Health, National University of Singapore, Singapore, Singapore.; ^3^Institute of Health Policy, Management and Evaluation, University of Toronto, Toronto, ON, Canada.; ^4^Office of the Permanent Secretary, Ministry of Public Health, Nonthaburi, Thailand.; ^5^School of Pharmaceutical Sciences, Universiti Sains Malaysia, Penang, Malaysia.; ^6^Department of Pharmacology and Clinical Pharmacy, Faculty of Pharmacy, Universitas Padjadjaran, Bandung, Indonesia.; ^7^HIV, Health and Development Team, United Nations Development Programme, Bangkok, Thailand.; ^8^Health Systems Research Institute (HSRI), Nonthaburi, Thailand.

**Keywords:** COVID-19, Regional Collaboration, Pandemic Preparedness

## Abstract

The coronavirus disease 2019 (COVID-19) pandemic has brought the need for regional collaboration on disease prevention and control to the fore. The review by Durrance-Bagale et al offers insights on the enablers, barriers and lessons learned from the experience of various regional initiatives. Translating these lessons into action, however, remains a challenge. The Association of Southeast Asian Nations (ASEAN) planned to establish a regional centre for disease control; however, many factors have slowed the realisation of these efforts. Going forward, regional initiatives should be able to address the complexity of emerging infectious diseases through a One Health approach, assess the social and economic impact of diseases on the region and study the real-world effectiveness of regional collaborations. The initiatives should seek to be inclusive of stakeholders including those from the private sector and should identify innovative measures for financing. This advancement will enable regions such as ASEAN to effectively prepare for the next pandemic.

 As the world enters the third year of the coronavirus disease 2019 (COVID-19) pandemic and continues to wrestle with uncertainties, there is an increasing understanding for the need for regional infectious disease control centres. However, there is a limited literature focusing on the initiation and operationalisation of such regional bodies outside Europe even though areas outside Europe are more vulnerable to emerging diseases, demonstrated by the empirical evidence on Ebola, Middle East respiratory syndrome (MERS), Swine flu, severe acute respiratory syndrome (SARS) and Avian flu.^1,2^ In addition, even before the pandemic, assessments indicated that low- and lower-middle income countries were less prepared to combat epidemics.^3^

 The scoping review by Durrance-Bagale et al on “Operationalising Regional Cooperation for Infectious Disease Control: A Scoping Review of Regional Disease Control Bodies and Networks” offers insights on the steps required to successfully initiate an infectious disease control body.^4^ The paper organises the lessons learned around seven dimensions on regional collaboration including organisational factors, effective networks, programming, diagnosis and detection, human resources, communication, and sustainability and funding. The review underscores the importance of taking contextual factors such as potential disease drivers, political-economy, socio-cultural, linguistic, geographical, and resources into account as well as having an open and inclusive conversation with relevant entities prior to establishing such a centre to discuss ideas, aims, opportunities, barriers, and ways of working. The study further calls for ensuring that human resources are strengthened through capacity-building and mentoring programmes, to reduce turnover and promote stability and sustainability in the organisational structure.

 This is a timely study as there are increasing demands for regional cooperation in Asia and elsewhere. The countries in the Association of Southeast Asian Nations (ASEAN), which are economically and socially integrated, have resolved to address cross-country management of infectious diseases and in late 2020, members announced the establishment of the ASEAN Center for Public Health Emergencies and Emerging Diseases (ACPHEED), outlining the scope of the initiative.^5,6^ The establishment of ACPHEED is a significant step towards promoting regional cooperation including in the field of virology research which could benefit from a centralised approach.^7^ Furthermore, regional coordination is critical for mitigating the impact of pandemics and minimising the economic shock at the domestic and regional levels.

## The Challenge of Translating Plans Into Action

 However, translating such a bold plan into action is not easy and there are challenges specific to the ASEAN region that impede the process. For one, ASEAN consists of ten extremely diverse member states, namely, Brunei, Cambodia, Indonesia, Lao PDR, Malaysia, Myanmar, the Philippines, Singapore, Thailand, and Vietnam. These countries have different types of political intuitions (democratic, socialist, military government); have different religions (Christianity, Islam, Buddhism); are at different levels of economic development (high-, middle- and low-income); have distinct demographic, geographic, and climatic characteristics; and have different types of health systems. This impacts the priorities of the countries as well as the ability to contribute to regional level initiatives. For example, surveillance and laboratory response, which has been identified as one of the areas for joint collaboration in ASEAN, would benefit from regional collaboration as capacity in member countries is varied.^7^ Table 1 lists few of the key characteristics of the ASEAN Member States.

**Table 1:** Selected Economic and Health System Characteristics of ASEAN Member States

**Country**	**ASEAN Membership Commencement Date**^a^	**GDP (Current, in Billion USD)**^b^	**GDP Per Capita (USD)**^b^	**Population ** **(in Millions)**^b^	**Health Expenditure (% of GDP)**^c^	**UHC Index**^d^
Brunei Darussalam	7-Jan-84	12.0	27 443.0	0.4	2.2	81
Cambodia	30-Apr-99	25.8	1543.7	16.7	7.0	60
Indonesia	8-Aug-67	1058.4	3869.6	273.5	2.9	57
Myanmar	23-Jul-97	79.9	1467.6	54.4	4.7	61
Lao PDR	23-Jul-97	19.1	2629.7	7.3	2.6	51
Malaysia	8-Aug-67	337.0	10 412.3	32.4	3.8	73
Philippines	8-Aug-67	361.5	3298.8	109.6	4.1	61
Singapore	8-Aug-67	340.0	59 797.8	5.7	4.1	86
Thailand	8-Aug-67	501.6	7186.9	69.8	3.8	80
Vietnam	28-Jul-95	271.2	2785.7	97.3	5.2	75

Abbreviations: ASEAN, Association of Southeast Asian Nations; GDP, gross domestic product; UHC, universal health coverage. Sources: ^a^About us, ASEAN. https://asean.org/about-us/. ^b^ Data as of 2020, World Development Indicators, The World Bank Group. https://data.worldbank.org/. ^c^ Data as of 2019, World Development Indicators, The World Bank Group. https://data.worldbank.org/. ^d^ Data as of 2017, UN Sustainable Development Goals. https://country-profiles.unstatshub.org/brn#goal-3.

 Second, there are institutional arrangements that impede effective management of the regional body. There have been several regional initiatives, however, as pointed out in Durrance-Bagale et al, these have been focused on specific activities and have been relegated to only one corporate function of ASEAN, rather than having a unified approach. Moreover, ASEAN’s health architecture is built on the principle of “non-interference,” which means regional agreements on health are based on building consensus across member states, each with a different interest and need, which can hamper collaboration.^8^ The effect of this arrangement is evident in it taking more than a year to identify a host country for ACPHEED; it is not expected that a host will be determined in the near future. Another consideration for the proposed regional body based on the dimensions indicated in Durrance-Bagale et al is that of funding. Funding support from the Government of Japan will be made available once a host is selected.^5^ However, it is not clear whether resources from within ASEAN have been identified for the purpose of this initiative, even as member states have pledged support, which will be relevant for the sustainability of the initiative.

 Third, while the ASEAN region is highly interconnected and has been impacted by the current pandemic, the regional, collective response has been minimal, even as there have been activities across countries in response to the pandemic.^9^ In the European Union and African Union, on the other hand, adoption of a regional approach to combatting the COVID-19 pandemic appears to have been helpful in mitigating its effects and has reinforced the imperative for regionalism in Europe and Africa.^10,11^ Beginning with a few Member States in ASEAN which adopted unilateral national responses during the very early phase of COVID-19 when cases were mainly imported through tourism, all ASEAN governments took a more nationalistic approach to respond to the pandemic, while focusing less on regional cooperation.^9,12^ Further, due to a lack of reliable estimates of a counterfactual scenario about what would have occurred if there was strong regional cooperation in ASEAN over the course of the pandemic, country governments have less incentive and justification for having stronger regional cooperation vis-à-vis their domestic priorities.

## The Way Forward

 As ASEAN and other regions take steps towards regional collaboration for pandemic preparedness, the findings of the review by Durrance-Bagale et al have several practical implications, few of which are reflected on here and summarised in Table 2.

**Table 2:** Summary of Challenges and Recommendations for Regional Collaboration in ASEAN, Based on Focus Areas Identified in Durrance-Bagale et al

**Focus**	**Challenges**	**Recommendations**
Organisational	Heterogenous composition of member countries, with differing objectives and capacities National priorities take precedence over regional collaborations Consensus based decision-making which can lead to delays in taking action	Generate evidence on the real-world effectiveness of regional cooperation for pandemic response
Networks	Several individual initiatives, however, focused on specific areas and not integrated with broader mandate of ASEAN	Inclusive stakeholder engagement, including private sector entities Recognise the complexity of infectious disease control, apply a One Health approach and consider infections in human as well as in animals when designing policies for disease prevention and control Expand networks from within health to non-health sectors
Sustainability and funding	Reliance on external sources of funding	Identify innovative financing mechanisms at the regional level Consider structural changes to financing mechanisms

Abbreviation: ASEAN, Association of Southeast Asian Nations.

 Technical analyses and consensus around common frameworks for research can strengthen the understanding and operation of regional collaborations. Currently, there is a lack of evidence on the real-world effectiveness of regional cooperation for pandemic response despite the belief that early policy reaction at the regional level will not only have a positive impact on flattening the pandemic curve but also on the response of the global value chain and domestic healthcare systems to the pandemic shock.^13^ Such technical analyses may be difficult to conduct and warrant further review as they could present evidence to governments to invest politically and financially in regional initiatives. It is important to recognise the complexity of infectious disease control and proactively attend to emerging issues. Notably, any regional initiative will need to apply a One Health approach and consider infections in human as well as in animals when designing policies for disease prevention and control.^14^ Further, regional initiatives should not only focus on the biomedical aspects of emerging diseases but also on their social and economic impact. For example, domestic activity across ASEAN countries has been less sensitive to the levels of infection rates and restrictions to mobility in comparison to the sharp contraction observed during the initial outbreak of COVID-19, in early 2020.^15^ Such changes in behavior will shape the policy response to the pandemic and therefore a holistic approach should be undertaken. This may include assessments of the labour market (for migrant workers, for example), international travel (eg, vaccination certificates) and trade (for personal protective equipment, pharmaceutical products and raw materials, among others). Organisationally, this will require a more cohesive approach to tackling infectious diseases.

 It is critical that regional initiatives are inclusive in terms of stakeholder participation. Such fora should not be limited to the public sector only: one of the chief lessons learned during the COVID-19 pandemic is that civil society, private sector (including pharmaceutical and medical device companies), and inter-governmental organisations play a crucial role in supporting the governments’ response. These actors can ensure public support and promote effective policy implementation as well as balance the health and economic priorities of disease control strategies in the region. It will also be important to raise awareness of the public regarding the benefits of regional cooperation, including for a potentially sensitive but important issue of sharing virus samples.^7^

 Another area highlighted in the review by Durrance-Bagale et al is that of financial sustainability of regional bodies. We need studies on innovative financing mechanisms such as taxes on digital platforms to support not only national bursaries but also for regional cooperation.^16^ The global debates on financing for the World Health Organization (WHO), whose role during the pandemic has become pronounced, also offers insights on the need for structural changes to financing mechanisms.^17^

## Conclusion

 The literature on regional international organisations and health is sparse and often normative. More studies on regional collaborations during COVID-19 are needed to inform about impact and give good and bad lessons to be learned by others. We encourage the *International Journal of Health Policy and Management* to continue to promote discussions on this important topic. The dreadful devastation caused by the COVID-19 pandemic offers a unique opportunity to propel regional collaborations and increase intra- and inter-regional cooperation in ASEAN and other regional organisations. Although it will take effort and political will, there is a real possibility that the ASEAN region will emerge stronger and better prepared for the next pandemic.

## Acknowledgements

 We thank the research team that conducted the review on “Operationalising Regional Cooperation for Infectious Disease Control: A Scoping Review of Regional Disease Control Bodies and Networks.” This study is supported by the Health Systems Research Institute (HSRI), Thailand.

## Ethical issues

 Not applicable.

## Competing interests

 Authors declare that they have no competing interests.

## Authors’ contributions

 YT conceptualized the paper and wrote the first draft with SVD. WI, TL, AsAS, AuAS, CO, JS, and NC provided critical inputs on the content of the manuscript.

## Disclaimer

 The findings, interpretations and conclusions expressed in this article do not necessarily reflect the views of the funding agencies.

## Funding

 The Health Intervention and Technology Assessment Program (HITAP), a semi-autonomous research unit in the Ministry of Public Health, Thailand, supports evidence-informed priority-setting and decision-making for healthcare. HITAP is funded by national and international public funding agencies. HITAP’s international work is supported by the International Decision Support Initiative, with the aim of providing technical assistance on health intervention and technology assessment to governments in low-income and middle-income countries. International Decision Support Initiative is funded by the Bill & Melinda Gates Foundation [OPP1202541], the UK’s Department for International Development, and the Rockefeller Foundation. HITAP is also supported by the Access and Delivery Partnership, which is hosted by the United Nations Development Programme and funded by the Government of Japan. HITAP is supported by the Health Systems Research Institute (HSRI), Thailand [grant number: HSRI.64-028] to conduct a situational assessment for the establishment of a Southeast Asia Center for Infectious Disease Control. HITAP is also collaborating with the Rockefeller Foundation to build capacity for infectious disease modelling to inform policy.
